# The differential effects of azithromycin on the airway epithelium in vitro and in vivo

**DOI:** 10.14814/phy2.12960

**Published:** 2016-09-21

**Authors:** Mariel Slater, Elizabeth Torr, Tim Harrison, Doug Forrester, Alan Knox, Dominick Shaw, Ian Sayers

**Affiliations:** ^1^ Division of Respiratory MedicineQueen's Medical CentreUniversity of NottinghamNottinghamUnited Kingdom

**Keywords:** Airway epithelium, asthma, azithromycin, barrier, matrix metalloproteinase‐9

## Abstract

Macrolides including azithromycin (AZM) can improve clinical symptoms in asthma regardless of infection status. The mechanisms underlying these beneficial effects are yet to be elucidated. The aim of this study was to determine the effect of AZM on the airway epithelial barrier both in an in vitro model and in patients with asthma. Primary human bronchial epithelial cells (HBEC) were grown at air liquid interface (ALI) and challenged using lipopolysaccharides from *Pseudomonas aeruginosa*. AZM was added at various stages and barrier integrity assessed using transepithelial electrical resistance (TEER) and permeability to FITC‐dextran. MMP‐9 levels were measured using ELISA. AZM enhanced barrier integrity (TEER/FITC‐dextran), increased thickness, suppressed mucin production, and MMP‐9 release during the formation of a normal epithelial barrier in vitro. MMP‐9 levels inversely correlated with TEER. AZM also enhanced maintenance of the barrier and facilitated repair post‐LPS challenge. To provide translation of our findings, 10 patients with moderate‐severe asthma were recruited and received 250 mg AZM o.d for 6 weeks. Bronchial biopsies taken pre‐ and post‐AZM treatment did not show evidence of increased epithelial barrier thickness or decreased mucin production. Similarly, bronchial wash samples did not show reduced MMP‐9 levels. Overall, our data show that AZM can significantly improve the development of a normal bronchial epithelial barrier in vitro, mimicking reepithelization postinjury. AZM also suppressed MMP‐9 release which correlated with barrier integrity, suggesting a putative mechanism. However, these effects were not observed in biopsy samples from asthma patients treated with AZM, possibly due to small sample size.

## Introduction

Azithromycin (AZM) is a broad‐spectrum antibiotic that can improve clinical outcomes in various respiratory diseases, regardless of infection status (Hahn et al. 2012; Spagnolo et al. 2013). Clinical benefits in asthma include improvements in: symptoms, peak expiratory flow, quality of life, and airway hyper‐responsiveness (AHR) (Reiter et al. 2013). Azithromycin may also prevent exacerbations in severe noneosinophilic asthma (Brusselle et al. 2013), chronic obstructive pulmonary disease (Albert et al. 2011), cystic fibrosis (CF) (Wolter et al. 2013), and non‐CF bronchiectasis (Wong et al. 2012). Mechanistic investigations of AZM report extended nonantibiotic properties including: antiinflammatory (Zimmermann et al. 2009), immunomodulatory (Shinkai et al. 2008) and antiviral actions (Gielen et al. 2010; Schogler et al. 2015), reduced mucin production (Ribeiro et al. 2009), and suppression of matrix metalloproteinases (MMP)‐2 and ‐9 (Murphy et al. 2007).

Asthma is a chronic inflammatory disease of the airways characterized by AHR and episodes of cough, breathlessness, and wheezing. It is thought that a dysfunctional epithelial lining of the airways is associated with the pathogenesis of asthma, leading to penetration of inhaled particles, bronchoconstriction, and inflammation (Holgate 2007). Chronic inflammation and bronchoconstriction are also thought to drive airway remodeling (Grainge et al. 2011), which may lead to fixed airflow obstruction. Therefore, treatments which enhance epithelial barrier integrity could help reduce infiltration of inhaled particles and inflammation leading to clinical benefit in asthma.

Modification of the bronchial epithelium has been previously reported in vitro*,* where addition of AZM to immortalized bronchial epithelial cell cultures influenced epithelial integrity (Asgrimsson et al. 2006). However, the effect of AZM on reepithelization, a key mechanism thought to be important in epithelial damage and repair, has not been investigated. Although macrolides improve symptoms and other measures of airway dysfunction in asthma (Reiter et al. 2013), the effect of AZM on structural cells of the airways and whether any effect on structural cells could be linked to clinical benefit, is unclear. Therefore, we hypothesized that AZM may enhance epithelial barrier integrity in vivo, contributing to the clinical benefits of AZM therapy observed in asthma.

The aim of this study was therefore to investigate the effects of AZM on human bronchial epithelial cell function in vitro and determine if these findings are also observed in the airways of asthma patients treated with AZM, that is, provide direct translation of our in vitro findings.

## Materials and Methods

### Cell culture

Normal healthy human bronchial epithelial cells (HBEC) (Lonza^®^ Clonetics^™^, Basel, Switzerland) and a bronchial brush sample from a healthy volunteer (see clinical study for ethics details) were grown at ALI (Stewart et al. 2012a,b). The cells were from three donors; all male, Caucasian, aged 19, 22, and 43 years. Briefly, cells were thawed and seeded into T75 cm^2^ flasks at a density of 3500 cells/cm^2^ in full growth media ([bronchial epithelial growth medium [BEGM, Lonza^®^], consisting of bronchial epithelial basal medium [BEBM, Lonza^®^]) plus SingleQuots^®^ bullet kit (13 mg/mL bovine pituitary extract, 2 mL 0.5 mg/mL hydrocortisone, 0.5 mL 0.5 *μ*g/mL hEGF, 0.5 mL 0.5 mg/mL epinephrine, 0.5 mL 10 mg/mL transferrin, 0.5 mL 5 mg/mL insulin, 0.5 mL 0.1 *μ*g/mL retinoic acid, 0.5 mL 6.5 *μ*g/mL triiodothyronine, 0.5 mL 50 *μ*g/mL gentamicin and amphotericin B, 0.5 mL). Fresh medium was added after 24 h and then every 2 days until 90% confluent. Media was changed after subculture to bronchial epithelial differentiation media (BEDM) consisting of 1:1 BEBM (Lonza):Dulbecco's modified eagle's media (DMEM) (Sigma‐Adrich^®^) + SingleQuots^®^ (Lonza^®^) excluding triiodothyronine and retinoic acid, with 50 nmol/L final concentration of retinoic acid supplemented at time of addition. Passage 3 cells were seeded into transwells (0.4 *μ*m pore size, 6.5 mm diameter, 0.33 cm^2^ growth area, Corning Life Science) at a density of 4 × 10⁴ cells in 200 *μ*L BEDM per transwell. When cells were confluent, BEDM was removed from the apical (upper) compartment, cells washed with warm sterile phosphate‐buffered saline (PBS), and media replaced in the basolateral (lower) compartment only from that time onwards. AZM dihydrate (Pfizer Inc., New York, NY) was dissolved in 100% ethanol to 12.5 mg/mL and further diluted in culture medium at time of addition. AZM, vehicle, or media was added to the basolateral chamber at various times during cultures.

### Transepithelial electrical resistance measurements

Transepithelial electrical resistance (TEER) was measured using the EVOM^2^ Voltohmeter with STX2 chopstick electrodes (World precision Instruments, Stevenage, UK) as directed by the manufacturer. Media was aspirated and cells were washed apically with PBS to remove mucus. A quantity of 200 *μ*L apical and 500 *μ*L basolateral BEDM was added to each transwell and cells were left to equilibrate at 37°C and 5% CO_2_ for 20 min before readings were taken. TEER measurements were corrected using transwell inserts with no cells (subtracted from each measurement) and values were corrected to filter size.

### Fluorescein isothiocyanate (FITC)‐dextran transport assay

Barrier permeability was measured by adding 200 *μ*L of 1 mg/mL 4 kDa FITC‐dextran (Sigma) diluted in bronchial epithelial basal cell media (BEBM, Lonza) to the apical surface of cells with 500 *μ*L basolateral BEBM. After 24 h, transwells were removed and basolateral supernatants were transferred to a clear bottom black 96‐well plate and measured in triplicate (485 nm excitation, 530 nm emission).

### Immunofluorescence

Transwells were washed in PBS, fixed in 4% formaldehyde, and stored at 4°C. Cells were blocked and permeabilized using 1% BSA +10% Goat serum in 0.15% Triton X‐100 in PBS for 1 h and then incubated with anti‐MUC5AC monoclonal IgG antibody (Sigma) overnight at 4°C. Secondary antibody was then added 1:100 (Goat anti‐mouse IgG, Alexfluor488 [Invitrogen/Life Technologies, Paisley, UK]) for 1 h at room temperature. Transwells were mounted with DAPI fluorescent mounting media (Vector Laboratories Ltd., Peterborough, UK) and left overnight at 4°C before analysis using a spinning disk confocal microscope with Volocity software (Version 5.5, PerkinElmer, Cambridge, UK).

### Clinical study

Ten participants with moderate‐severe asthma were recruited in this study (Tables 1 and 2), which was performed in accordance to the Declaration of Helsinki with ethical approval (reference 11/EM/0062), Clinical Trials Register EudraCT: 2011‐000237‐36. Participants gave written consent for clinical assessments and bronchoscopies both pretreatment (visit 1) and posttreatment (visit 2), following 250 mg daily open‐label AZM (Zithromax, Pfizer Inc.) for 6 weeks. Patients had no clinical sign of infection and had not received antibiotics for 6 weeks.

**Table 1:** Inclusion and exclusion criteria for azithromycin in asthma (AZA) clinical study

Inclusion Criteria	Exclusion criteria
Male or female aged between 18 and 80 years old Clinical diagnosis of refractory asthma Symptomatic despite receiving treatment at step 4 of the BTS asthma guidelines: evidence of poor asthma control in terms of regular night‐time awakening (>2/week) or more than four puffs of relief medication/day (>twice/week) requiring repeated (two or more per year) courses of oral corticosteroids despite treatment with high‐dose inhaled corticosteroids (≥1000*μ*g beclomethasone or equivalent) and treatment with, or a previous unsuccessful trial of, a long‐acting beta‐agonist or leukotriene antagonist	Pregnant females Inadequate contraception or lactation Antibiotic course within the last 6 weeks Smoking history in excess of 20 pack years Clinical diagnosis of allergic bronchopulmonary aspergillosis Bronchiectasis Abnormal liver function tests History of liver disease Medication known to interact with azithromycin (e.g., ciclosporin, digoxin, ergot derivatives, terfenadine, warfarin, antacids, and ritonavir)

**Table 2:** Asthma patient demographics at recruitment

Subject	Age	Gender	Other morbidities	Current treatments	Juniper ACQ score	% predicted FEV_1_
01	51	M	Sinus polyps	Carbicysteine, Salbutamol, Symbicort	0.17 (+)	53
02	48	F	Pacemaker	Phyllocontin, Salbutamol, Singulair, Symbicort	4.17 (−)	64
03	63	M	Sinusitis	Prednisolone, Salbutamol, Seretide	0.67 (+)	54
04	58	F	None	Phyllocontin, Prednisolone, Singulair, Symbicort, Terbutaline	3.67 (−)	58
05	20	F	None	Atrovent, Phyllocontin, Prednisolone, Salbutamol, Seretide, Tiotropium	2.67 (−)	81
06	62	M	Gout, sinus polyps	Salbutamol, Seretide, Singulair	1.17 (+/−)	80
07	57	F	Diabetes	Salbutamol, Seretide, Singulair	2.00 (−)	90
08	19	F	None	Singulair, Symbicort	0.50 (+)	67
09	36	F	None	Phyllocontin, Prednisolone, Symbicort	1.67 (−)	90
10	46	F	Depression	Fostair	2.50 (−)	43

Ten subjects with asthma were recruited meeting inclusion criteria. F = female/M = male, ACQ, asthma control questionnaire; (+), adequate asthma control according to ACQ scores (≤0.75); (−), inadequate asthma control (≥1.5); (+/−), the cross‐over between adequate and inadequately controlled asthma; FEV_1_, forced expiratory volume in 1 sec.

### Bronchoscopy procedure

Endobronchial biopsies were obtained using standard procedures (du Rand et al. 2013; Shaw et al. 2015). Samples from asthma patients both pretreatment (visit 1) and posttreatment (visit 2) were collected.

### Hematoxylin and eosin (H&E) staining and assessment of barrier thickness

Transwells containing cells treated for 14 days ±AZM were washed in media and then 3 × 15 min in PBS. These were then fixed in 4% formaldehyde and paraffin embedded. Mounted sections of biopsies or transwells were sectioned and stained with H&E or alcian blue. Mounted sections were imaged using a Nikon light microscope with 40× objective Nikon UK Limited, Kingston upon Thames, UK. Barrier thickness was assessed in four separate wells per condition from two different HBEC donors (*n *=* *4) or all biopsies available. Three images were taken from three different sections and three measurements were made at one quarter, one half, and three‐quarter intervals by setting a scale using the width of the transwell filter (10 *μ*m) with Image J software (Version1.45s, National Institute of Health, Bethesda, MD). Cell nuclei were counted in each image and expressed as cells per 100 *μ*m length of transwell.

### MMP‐9 measurements

Active MMP‐9 was measured using a Fluorokine^®^ E assay (R&D Systems, Abingdon, UK) following manufacturer recommendations. MMP‐9 was normalized to total protein in bronchial washes via Bradford assay.

### Statistical analyses

PRISM software (Version6.03, GraphPad Software Inc., La Jolla, CA) was used for statistical analyses. Two‐way ANOVA compared differences over time compared with controls. All data were analyzed using nonparametric analyses: Wilcoxon sign‐rank test for paired and Mann–Whitney test for unpaired analyses and Kruskal–Wallis test for multiple comparisons. Spearman's correlation assessed the relationship between outcome measures. A *P *<* *0.05 was considered significant.

## Results

### AZM improves barrier formation in differentiating bronchial epithelial cells

**Figure 1:**
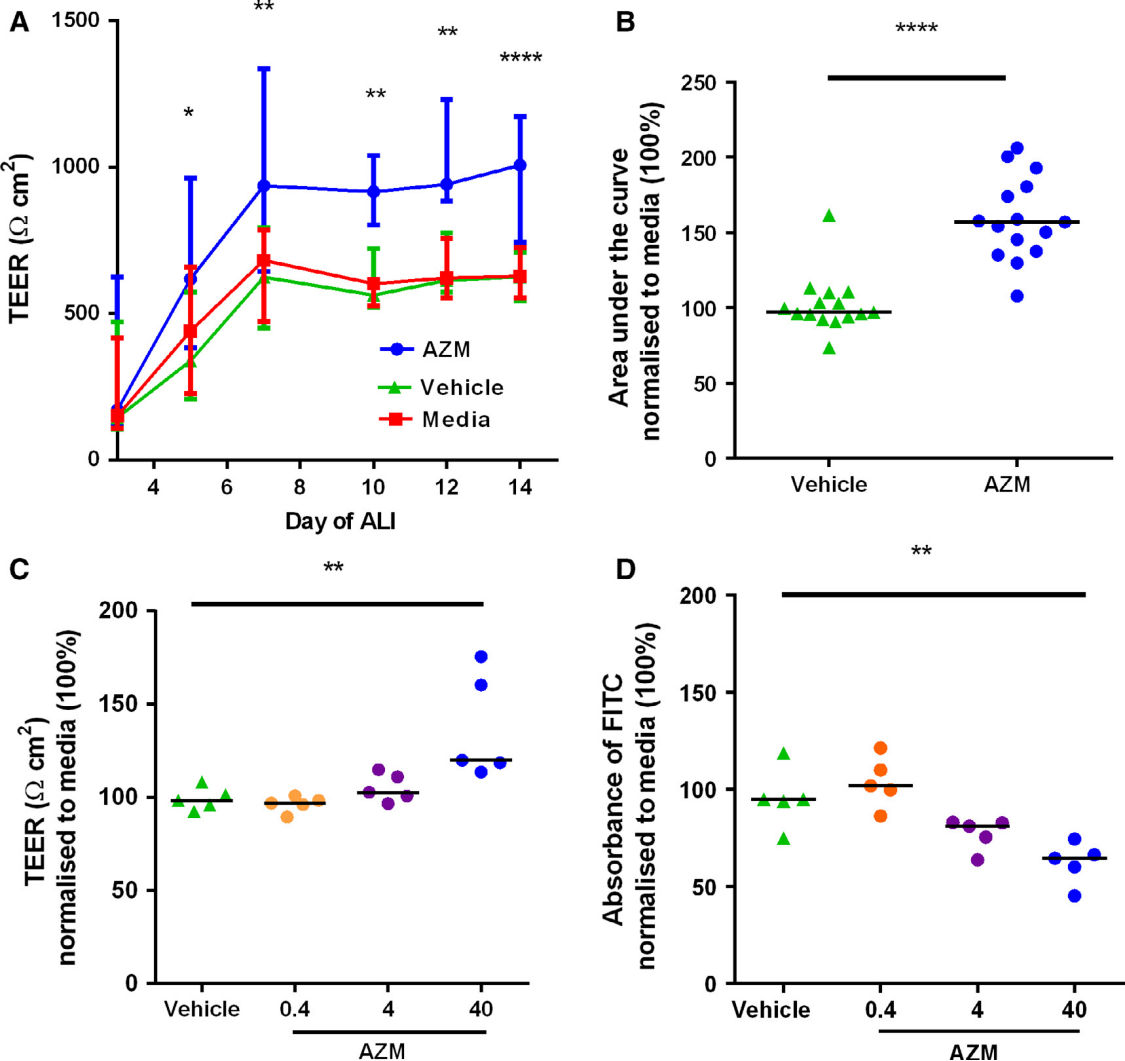
Azithromycin improves airway epithelial barrier formation in vitro. (A) Transepithelial electrical resistance (TEER) of media, vehicle, and 40 *μ*g/mL azithromycin (AZM)‐treated human bronchial epithelial cells (HBEC) at air liquid interface (ALI) over 14 days (*n = *15). (B) Area under the curve analysis of (A). (C) TEER of vehicle, AZM (0.4–40 *μ*g/mL)‐treated HBEC at ALI day 14 alone (*n *=* *5). (D) Absorbance of fluorescein isothiocyanate (FITC)‐dextran from HBEC in figure (C). Figure A shows median (+/−IQR). Results from (B, C, and D) were normalized to media (100%) in each experiment. Experiments from three different HBEC donors. *P <* *0.05; **P <* *0.01; ****P <* *0.0001.

To mimic bronchial reepithelization, we examined the effect of AZM during differentiation of primary HBEC at ALI (Fig. 1). AZM enhanced the formation of a barrier as assessed by both TEER (Fig. 1A, B, and C) and passage of FITC‐dextran (Fig. 1D). AZM (40 *μ*g/mL) treatment significantly elevated TEER by day 14 (*n *=* *15, *P *<* *0.0001) (Fig. 1A). Area under the curve (AUC) analysis normalized to media (100%) confirmed this finding, where AZM = 157% (138–181) versus vehicle = 97% (94–110) (15 experiments in three donors, *P *<* *0.0001) (Fig. 1B). However, in titration studies, 4 or 0.4 *μ*g/mL had no effect on TEER (Fig. 1C). The higher dose of AZM (40 *μ*g/mL) significantly reduced passage of FITC‐dextran compared with vehicle, where absorbance normalized to media (100%) in AZM‐treated cells = 65% (53–71) versus vehicle‐treated cells = 95% (84–107) (*P *=* *0.002). The lower doses of AZM were not statistically different to vehicle, suggesting a dose–response effect (Fig. 1D). There was a significant correlation between TEER and FITC‐dextran permeability data in each experiment (correlation (*r*) range = −0.51 to −0.79, *P* < 0.001), which has been reported previously (Xiao et al. 2011).

### AZM increases epithelial barrier thickness

**Figure 2:**
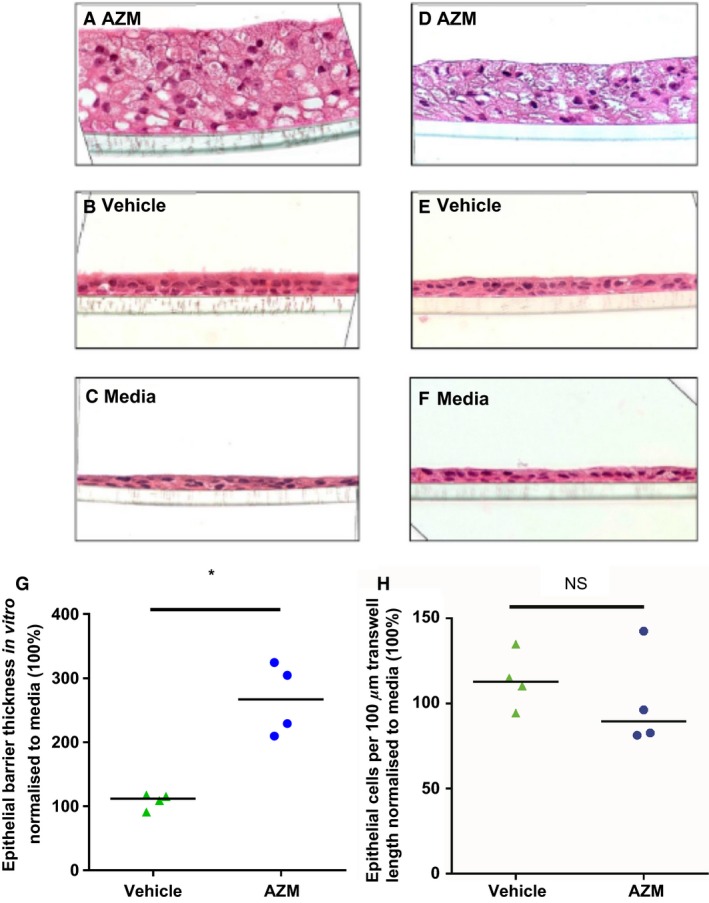
Azithromycin increases airway epithelial layer thickness in vitro. Azithromycin (AZM) treatment from air liquid interface (ALI) day 0 generates a visibly thicker epithelial layer by day 14. Cells were treated for 14 days (3× weekly) at ALI with 40 *μ*g/mL AZM, vehicle, or media, at which time they were fixed and stained with hematoxylin and eosin. HBEC donor 1 treated with (A) 40 *μ*g/mL AZM, (B) vehicle, and (C) media. HBEC donor 2 treated with (D) 40 *μ*g/mL AZM, (E) vehicle, and (F) media. Representative light microscope images (×40 objective) from four wells per condition. (G) Median epithelial barrier thickness of HBEC layer at ALI day 14 in cells treated with 40 *μ*g/mL AZM, vehicle, or media (thickness compared with media (100%) from each experiment, (*P* = 0.014, *n* = 4). (H) Cell counts per 100 *μ*m transwell length were normalized to media (100%) (*P* > 0.05, *n* = 4). NS, nonsignificant.

To identify morphological changes induced by AZM on the epithelial barrier in vitro, we measured barrier thickness in ALI sections (Fig. 2). AZM treatment leads to the formation of a thicker cellular layer in both donors, indicating alterations in the morphology of the epithelial barrier (Fig. 2A–F). Median barrier thickness of cells treated with AZM, vehicle, or media alone for 14 days with IQR: AZM = 22.0 *μ*m (19.1–26.3), vehicle = 9.2 *μ*m (8.1–10.2), and media = 8.8 *μ*m (7.7–9.0) (*n *=* *4, *P *=* *0.02). When normalized to media from each experiment (100%), barrier thickness of cells treated with AZM = 266% (214–319) versus vehicle = 112% (95–117) (*n *=* *4, *P *=* *0.014) (Fig. 2G). To further define the factors underlying this increased barrier thickness in AZM‐treated cells, that is, hyperplasia versus hypertrophy, we determined cell numbers per 100 *μ*m on these transwell images. There was no significant increase in cell number in the AZM‐treated epithelial layers (Fig. 2H).

### AZM suppresses mucin production

**Figure 3:**
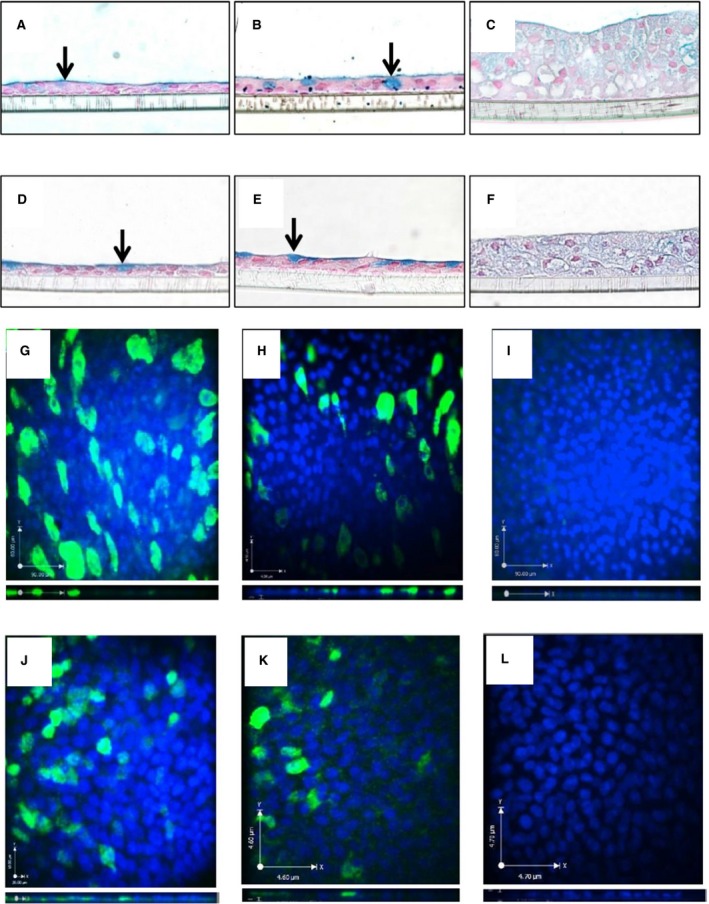
Azithromycin suppresses mucin production by airway epithelial cells in vitro. The effect of 40 *μ*g/mL azithromycin (AZM), vehicle, and media on alcian blue glycoprotein staining of ALI day 14 HBEC. Cells were treated 3× weekly for 14 days at ALI and transwells were fixed, sectioned, stained, and imaged using a light microscope (40× objective). HBEC donor 1 treated with (A) media, (B) vehicle, and (C) AZM. HBEC donor 2 treated with (D) media, (E) vehicle, and (F) AZM. Arrows point to dark blue staining, suggesting mucus‐secreting goblet cells. Confocal microscopy of mucin 5AC (MUC5AC) immunofluorescence of HBEC treated for 21 days at ALI for donor 1: (G) medium, (H) vehicle, and (I) 40 *μ*g/mL AZM and donor 2: (J) medium, (K) vehicle, and (L) 40 *μ*g/mL AZM.

To complement and extend previous evidence that AZM suppresses mucin production, we investigated mucin expression in ALI sections using alcian blue staining and MUC5AC immunofluorescence (Fig. 3). AZM treatment resulted in the formation of an epithelial barrier with reduced and nonuniform mucin expression, as assessed by alcian blue staining of transwells after 14 days ±AZM treatment (Fig. 3A–F). Similarly, MUC5AC immunofluorescent staining of ALI day 21 cells in this model confirmed suppression in AZM‐treated cells (Fig. 3G–L).

### AZM maintains an established bronchial epithelial cell barrier

**Figure 4:**
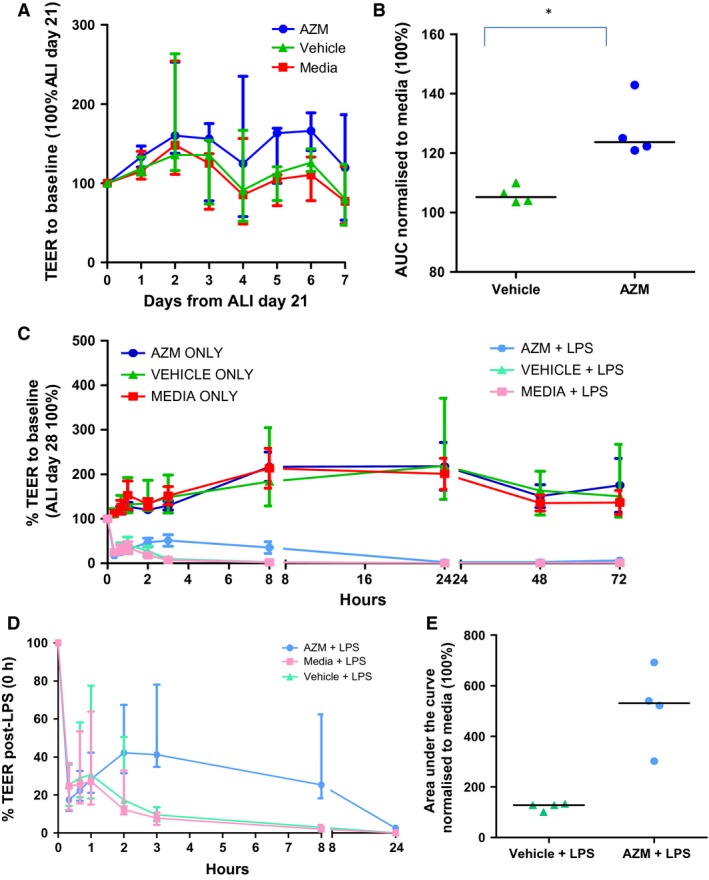
Azithromycin promotes maintenance and repair of an airway epithelial barrier in vitro. (A) TEER values of cells treated with azithromycin (AZM) (40 *μ*g/mL), vehicle, or media daily from ALI day 21–28 normalized to individual values at baseline (100%, day 21) (*n *=* *4, median with interquartile range). (B) Area under the curve analysis (of A) normalizing data to media (100%) in each experiment, with median (*n *=* *4, median with IQR). *P <* *0.05. HBEC were pretreated for 7 days with 40 *μ*g/mL AZM, vehicle, or media alone and then challenged apically with 1 mg/mL LPS or media alone (unchallenged) (*n = *4), (C) TEER over 72‐h post‐LPS addition (day 28) normalized to baseline value (0 h), (D) TEER of cells pretreated for 7 days with AZM (40 *μ*g/mL), vehicle, or media and challenged with 1 mg/mL LPS at ALI day 28 for 24 h, normalized to prechallenged TEER (magnified as part of Figure Panel C) (*n = *4, median with IQR). (E) Area under the curve analysis of (F) normalized to media (100%), with median values shown. *P <* *0.05; **P <* *0.01; ***P <* *0.001.

To determine the effect of AZM on barrier integrity when a differentiated barrier had been established, cells were differentiated at ALI for 21 days. AZM was then added daily for 7 days and TEER measurements were recorded (Fig. 4A). AZM aids in barrier maintenance: median TEER (AUC) days 21–28 with IQR, AZM = 124% (121–138) versus vehicle = 105% (104–109) (*n *=* *4, *P *=* *0.03) (Fig. 4B).

### AZM pretreatment facilitates the acute response to bronchial epithelial cell damage

To assess the response of differentiated cells to damage, potentially mimicking respiratory infection, we investigated the effect of AZM pretreatment on cell response to LPS (*P. aeruginosa)*. Cells grown to ALI day 21 were pretreated ±AZM for 7 days, then 1 mg/mL LPS was added apically for 24 h. TEER was read at intervals up to 72 h. LPS addition led to a significant loss in epithelial integrity as determined by a drop in TEER for all treatment groups (Fig. 4C). However, AZM (40 *μ*g/mL) pretreated cells demonstrated a reestablishment of TEER at 3 h compared with vehicle (*n *=* *4, *P *<* *0.01) (Fig. 4D). Median TEER with IQR normalized to media over 24 h; AZM = 531% (357–654) and vehicle control = 128% (107–132) (*n = *4, *P *=* *0.03) (Fig. 4E). At 24 h, this protective effect of AZM was no longer present and all treatment groups demonstrated minimal TEER. TEER of unchallenged controls did not drop below baseline levels (Fig. 4C).

### AZM suppresses MMP‐9 levels

**Figure 5:**
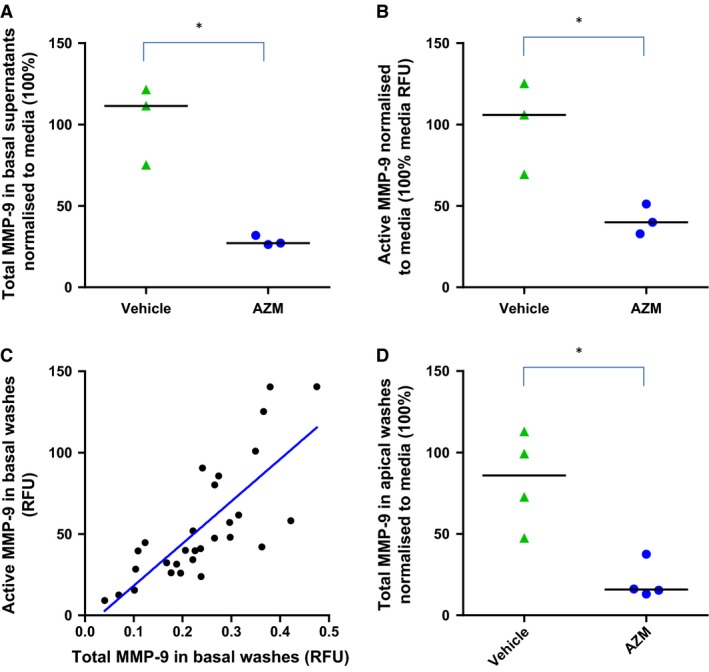
Azithromycin suppresses both total and active MMP‐9 production by airway epithelial cells in vitro. MMP‐9 from cells treated ±azithromycin (AZM) (40 *μ*g/mL, 3× weekly) at ALI day 14. (A) Basolateral total MMP‐9. (B) Basolateral active MMP‐9. (C) Total versus active basolateral MMP‐9 levels show a highly correlated expression, *r* = 0.84, *P* < 0.0001. (D) Apical wash total MMP‐9. Data from (A, B, C) normalized to media (100%) with median. Absolute total MMP‐9 range: 0.12–36.4 ng/mL. Three to four experiments (four wells per condition). *P <* *0.05.

To assess the effect of AZM on MMP‐9, levels of both total and active MMP‐9 in basolateral supernatants from ALI experiments pretreated for 14 days ±AZM were measured (Fig. 5A and B). Both total and active MMP‐9 were significantly suppressed in these basolateral supernatants following treatment with AZM (40 *μ*g/mL). Total MMP‐9 (median relative fluorescence unit [RFU]) with IQR normalized to media; AZM = 32% (31–36) and vehicle control =101% (77–110) (*n *=* *3, *P *=* *0.050) and active (median RFU) with IQR normalized to media; AZM = 50% (34–51) and vehicle control = 111% (88–130) (*n *=* *3, *P *=* *0.050). There was a significant correlation between total and active MMP‐9 in these samples (*r* = 0.84, *P *<* *0.0001) (Fig. 5C). Similar findings were observed in apical wash samples where total MMP‐9 concentrations were significantly suppressed following AZM treatment; AZM (median, IQR) = 16% (14–32) and vehicle control = 86% (54–109) (*n *=* *4, *P *=* *0.012, Fig. 5D).

### MMP‐9 levels and barrier thickness correlate with epithelial integrity

**Figure 6:**
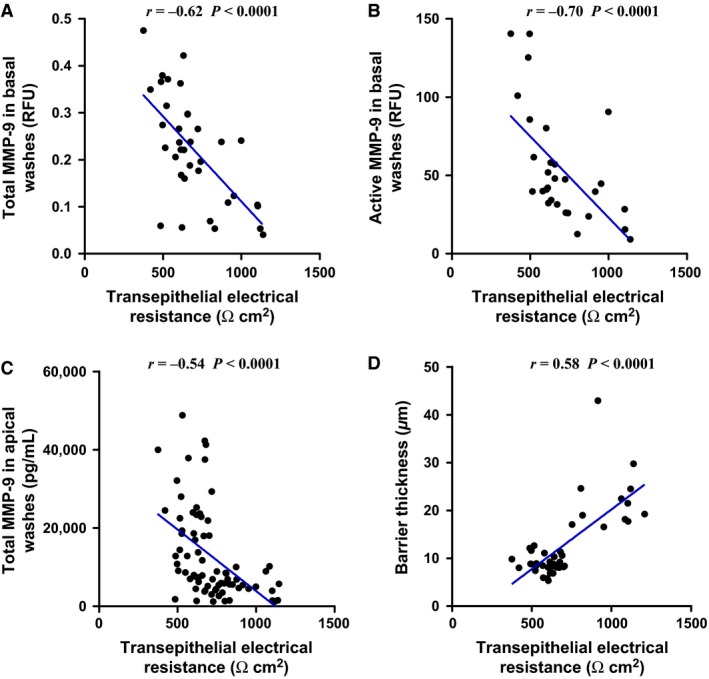
TEER correlates with MMP‐9 levels and barrier thickness. TEER correlation with (A) total and (B) active MMP‐9 levels in basolateral supernatants at ALI Day 14, (C) total MMP‐9 in apical washes in cells at ALI Day 14, and (D) barrier thickness at ALI day 14. Data presented is from individual well and includes all treatments.

In order to translate our morphological and secretory findings that AZM significantly suppresses MMP‐9 and also leads to a significant increase to epithelial barrier thickness, we correlated these outcomes with our primary measure of epithelial integrity, TEER (Fig. 6). These data demonstrate that total basolateral MMP‐9 (*r* = −0.62 *P* < 0.0001), active basolateral MMP‐9 (*r* = −0.70 *P* < 0.0001), and total apical MMP‐9 (*r* = −0.54, *P *<* *0.0001) all negatively correlated with TEER (Fig. 6A–C). Similarly, epithelial barrier thickness was positively correlated with TEER (*r* = 0.58, *P *<* *0.0001) (Fig. 6D).

### Asthma patient studies do not translate in vitro findings

**Figure 7:**
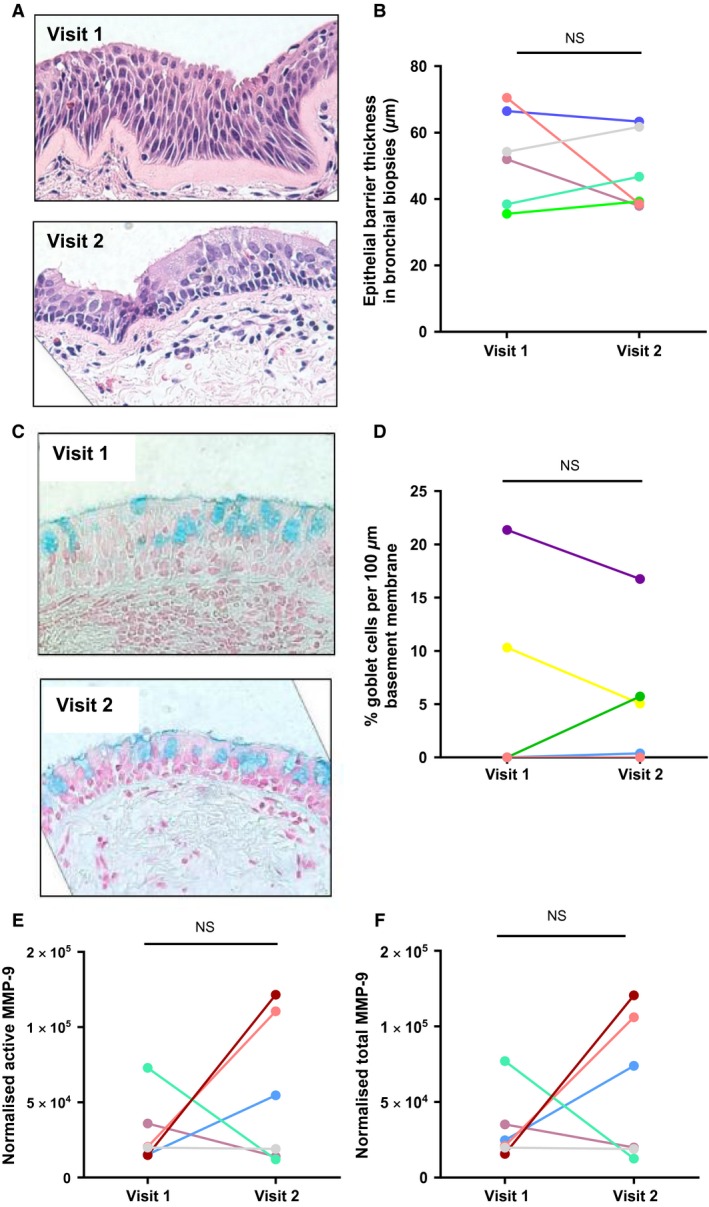
Azithromycin has heterogeneous effects on bronchial epithelial cells in vivo. Ten moderate‐severe asthma patients were recruited and administered with 250 mg daily open‐label azithromycin (AZM) for 6 weeks (see methods). Bronchial biopsies were obtained from the right bronchus intermedius (RBI) and bronchial washes were taken from the right upper lobe (RUL) both pre‐ (Visit 1) and post‐AZM (Visit 2). (A) Representative images of bronchial biopsies collected from an asthma patient at visit 1 and 2 stained with hematoxylin and eosin, (B) Comparison of epithelial barrier thickness in bronchial biopsies at visit 1 and 2 (*n* = 6), (C) Representative images of bronchial biopsies collected from an asthma patient at visit 1 and 2 stained with alcian blue, (D) Comparison of % goblet cells/100 *μ*m basement membrane in bronchial biopsies at visit 1 and 2 (*n* = 6). Total and active MMP‐9 in wash supernatants normalized to total protein, (E) Corrected active MMP‐9 in wash samples at visits 1 and 2, and (F) Corrected total MMP‐9 in wash samples at visits 1 and 2. NS, nonsignificant (*P* > 0.05).

In order to translate our in vitro finding, we investigated the effect of AZM on the airway epithelium in vivo by recruiting 10 asthma patients to complete a clinical study involving 6 weeks of AZM treatment. A bronchial biopsy at visit 1 (pre‐AZM) and visit 2 (6 weeks post‐AZM) was collected. Biopsy morphology varied significantly between patient/visit emphasizing the heterogeneity of asthma observed in other studies (see representative Fig. 7A). Overall, epithelial barrier thickness of asthma patient biopsies (six per visit) did not alter post‐AZM; visit 1 = 53.1 *μ*m (35.5–70.5) versus visit 2 = 43.0 *μ*m (37.9–63.3) (*P* = 0.675) (median and range). There was also no significant difference in epithelial thickness within each patient between visits (Fig. 7B). Biopsies from asthma patients were also stained with alcian blue and goblet cells were counted (Fig. 7C). Goblet cell number varied between patient/visit: statistical analysis of five biopsies at two visits revealed no significant difference between % goblet cells at visit 1 (0.0% [0.0–15.8]) versus visit 2 (5.1% [0.2–11.2]), either between groups (*P* = 0.683) or within each patient (*P* > 0.999) (median and range) (Fig. 7D). MMP‐9 levels were also measured in bronchial washes pre‐ and post‐AZM treatment. Analyses revealed no significant difference between active MMP‐9 within each patient between visits (Fig. 7E and F). Furthermore, MMP‐9 levels did not correlate with epithelial biopsy thickness (data not shown). Finally, we also determined if AZM resulted in hyperplasia in vivo to complement our in vitro analyses; there was no significant difference in cell counts (per 100 *μ*m basement membrane length) in biopsies from asthma patients between visit 1 (35.9 [31.2–47.4]) and visit 2 (39.8 [29.3–48.7]) (*P* = 0.788) (median and range). Pairwise analysis confirmed no significant differences in cell counts between visits (*P* = 0.688, data not shown).

## Discussion

Macrolide antibiotics, including AZM, have additional properties beyond those of an antimicrobial and show potential utility in asthma and other airway diseases (Richeldi et al. 2008; Hernando‐Sastre 2010). However, an underlying mechanism of action is yet to be defined. In this study, we further define the effects of AZM on the bronchial epithelial cell barrier, a substratified layer of cells that is thought to be altered in asthma, using a physiologically relevant in vitro model. Our data demonstrate that AZM has multiple beneficial effects on epithelial barrier properties including: (1) improved barrier formation during differentiation, (2) improved maintenance of an established barrier, and (3) improved response of the epithelium to damage. Importantly, alterations in barrier properties were functionally relevant as assessed by permeability assays and morphological studies where AZM‐treated epithelial cell layers displayed hypertrophy, resulting in increased barrier thickness. AZM altered the expression profile of the epithelial layer including suppression of mucins and MMP‐9 expression. Endogenous release of MMP‐9 correlated with our primary measure of epithelial integrity (TEER), providing a putative mechanism. In contrast, we did not observe significant effects on the same series of outcomes in the bronchial epithelium in vivo using samples collected pre‐ and post‐AZM treatment in moderate‐severe asthma patients. Overall, these data provide novel insight into the additional properties of AZM on bronchial epithelial homeostasis, which is particularly relevant for the mechanism of action of AZM and derivatives in respiratory disease. In addition, this study highlights the challenge of translating in vitro findings to the more heterogeneous in vivo setting.

There are intrinsic differences between normal and asthmatic airway epithelium including: altered proliferation, cytokine and mucus production, susceptibility to injury, and the ability to develop a functional epithelial barrier (Kicic et al. 2006; Xiao et al. 2011). It has also been reported that tight junction protein (TJP) ZO‐1 and E‐cadherin are expressed at lower levels in asthmatic airways ex vivo (Holgate 2007) and that TEER is significantly lower in ALI cultures of asthma patient cells (Xiao et al. 2011). Therefore, improvement of the bronchial barrier may be a potential target for clinical benefit. We hypothesized that AZM may influence airway epithelial function as part of the beneficial effect observed in vivo, in particular, the ability of bronchial epithelial cells to develop, maintain, and repair a functional barrier. AZM enhanced the integrity of a developing HBEC barrier with a significant increase in TEER, barrier thickness, and a reduction in permeability to FITC‐dextran. There was no effect on cell number in ALI cultures compared to transwell length indicating that AZM treatment causes HBEC dysplasia or metaplasia. These data, to the best of our knowledge, provide the first evidence supporting an effect of AZM on reepithelization of a developing epithelial barrier. This information may be clinically relevant, that is, AZM may have greater benefits in severe asthma where exacerbation and epithelial shedding/reepithelization/differentiation are an ongoing process. Similarly, our observation of improved epithelial integrity post‐AZM in vitro has the potential to “correct” the intrinsic defects described in asthma patient cells observed at ALI (Xiao et al. 2011). However, it is important to be cautious in the interpretation of these in vitro findings and it remains unclear if the modest effects on barrier properties (TEER) we observed in cells isolated from subjects without respiratory disease are reproducible in cells from, for example, asthma patients. Similarly, it remains unclear if these in vitro measures truly relate to clinically relevant outcomes in patients. Another consideration is the dramatic change in morphology of the epithelial barrier induced by AZM, that is, increased thickness. It is feasible that this anatomical change may actually be detrimental to airway function. These considerations were the foundation for an asthma patient arm to this study.

In addition to improving the formation of a barrier, AZM also promoted the maintenance of an already established epithelial barrier. Similar improvements in TEER have been demonstrated using the same dose of AZM (40 *μ*g/mL) in differentiated immortalized HBEC, with no effect on cell proliferation, viability, or apoptosis. AZM treatment also caused internalization of TJP occludin and claudins (Asgrimsson et al. 2006). The current and previous findings have clear inferences for the preservation of epithelial barrier integrity. We also used LPS from *P. aeruginosa* to mimic respiratory tract infection leading to epithelial damage. AZM did not protect against the initial challenge, though results indicate that AZM helps reestablish barrier integrity compared with vehicle, shown by increased TEER between 2 and 3 h postchallenge which was maintained to at least 8 h. This information suggests that AZM pretreatment primes cells to respond to injury, supporting low‐dose long‐term therapy which could speed up patient recovery postexacerbation. It is important to note that this reestablishment of TEER was lost at 24‐h postinjury, indicating an acute phase response which may be dependent upon challenge duration. Our data are in good agreement with the recent finding that 60 *μ*g/mL AZM was protective in human gingival epithelial cells when challenged with TNF*α*, including the maintenance of epithelial barrier (TEER) (Miyagawa et al. 2016). This study went on to suggest that the mechanism underlying this effect, at least in part, involved AZM inhibiting the phosphorylation of ERK and p38 MAP kinase in TNF*α*‐treated cells and preventing a decrease in E‐cadherin (Miyagawa et al. 2016). A limitation of this study is that we did not evaluate the effect of AZM on these and other signaling pathways in the different stages of the experiments, however, Miyagawa and colleagues did not relate these signaling changes to the primary outcome TEER leaving findings difficult to interpret (Miyagawa et al. 2016). One hypothesis that we evaluated was the potential role of MMP‐9 in these responses.

MMP‐9 is secreted by a host of cells including neutrophils, eosinophils, dendritic cells, and epithelial cells and acts on fibrin, denatured collagen (gelatin), and TJP ZO‐1 as well as other substrates (Demedts et al. 2005). MMP‐9 is elevated in severe acute asthma (Lemjabbar et al. 1999; Belleguic et al. 2002), during exacerbations of asthma (Oshita et al. 2003), and postallergen challenge (Kelly et al. 2000). MMP‐9 gene deletion attenuates allergic asthma in a mouse model (Vermaelen et al. 2003) further suggesting its role in asthma and airway remodeling. MMP‐9 specifically deregulates TJP and increases the permeability of macromolecules in differentiated HBEC (reversible on tissue inhibitor of metalloproteinase [TIMP]1 addition) (Vermeer et al. 2009). Macrolides are known to suppress MMPs at both the gene and protein levels both in vitro and in vivo (Simpson et al. 2008; Ribeiro et al. 2009; Verleden et al. 2011). Therefore, we hypothesized that MMP‐9 suppression by AZM in our experiments may at least in part underlie the beneficial effects observed on integrity. We reproduced and extended previous work to show that AZM suppresses both total and active MMP‐9, but demonstrate for the first time that endogenous release of MMP‐9 directly correlates with barrier integrity, providing a putative mechanism. While a limitation of this study was that we did not add exogenous MMP‐9 in an attempt to reverse the beneficial effects of AZM, others have shown that recombinant MMP‐9 added to the epithelial ALI model results in a significant loss of epithelial integrity (measured by TEER), TJP localization, and permeability to macromolecules (Vermeer et al. 2009). Importantly, MMP‐9 suppression has been demonstrated in vivo in BAL samples taken from lung transplant patients post‐AZM treatment supporting our in vitro observations (Verleden et al. 2006). The finding that AZM suppresses MMP‐9 has implications for targeting treatment to multiple lung conditions and warrants further investigation both in vitro and in vivo.

In bronchial epithelial cells cultured from asthma patients, there is a reported increase in mucin production (Holgate 2007), including MUC5AC. Our data support previous findings (Ribeiro et al. 2009) demonstrating that HBEC pretreated with AZM for 14 and 21 days results in a complete suppression of mucin production leading to negligible staining with alcian blue and MUC5AC compared with controls. Mucus is required for clearance of inhaled particles in the lung, but can exacerbate airway obstruction; this may be important in fatal asthma where mucus plugging has been described (Bai and Knight 2005). Therefore, in addition to the beneficial effects of AZM on epithelial integrity and MMP‐9, mucin suppression is also considered a clinically relevant property. While we did not define the mechanism underlying the suppression of MUC5AC by AZM, a recent study has shown that AZM can suppress MUC5AC (and MUC2) in HBECs stimulated with IL‐13 at the mRNA and protein levels and suggested a mechanism involving chloride channel accessory 1 (CLCA1) (Mertens et al. 2016). CLCA1 has been shown to be elevated in the airway epithelium in asthma (Woodruff et al. 2009) and linked to MUC5AC expression (Kim et al. 2007; Mishina et al. 2015). A role for AZM suppression of extracellular signal‐regulated kinase (ERK) 1/2 and I‐kappa B phosphorylation has also been suggested to contribute to MUC5AC suppression (Imamura et al. 2004). Overall, these data suggest that further investigation of the effect of AZM on mucin production and also goblet cell hyperplasia and metaplasia are warranted with potential implications for multiple diseases where mucus hypersecretion is a feature.

While our in vitro data are compelling regarding the additional properties of AZM on bronchial epithelial cell homeostasis including barrier properties and secretory profile, we set out to translate these finding to the in vivo situation. Our initial aim was to recruit 10 eosinophilic asthma patients and 10 neutrophilic asthma patients, based on the ability to detect a 0.5 difference in the Juniper asthma control score (ACQ), with 80% power. However, we could not recruit to target and we had a significant drop out rate as not all patients tolerated the first bronchoscopy. This left 10 asthma patients to complete a clinical study involving 6 weeks of AZM treatment with six patients providing both pre‐ and post‐AZM samples for analyses. Examination of bronchial biopsies from 10 moderate‐severe asthma patients treated 250 mg daily open‐label AZM (Zithromax, Pfizer Inc.) for 6 weeks did not identify any significant change in: (1) bronchial epithelial cell thickness, (2) mucin production (% goblet cells), and (3) MMP‐9 production (bronchial wash) when pre‐ and post‐AZM samples were compared. Data generated for each of these outcomes were highly heterogeneous, potentially as anticipated for an in vivo study using relatively small numbers of recruited patients which may have limited our ability to observe measurable effects of AZM.

Similarly, it is difficult to compare the in vitro situation where bronchial epithelial cells will have a sustained AZM exposure in contrast to the in vivo epithelium were exposure is difficult to evaluate. A key limitation of our study was that we used cells from subjects without respiratory disease (e.g., not asthma patients) to investigate AZM in vitro and then used asthma patients to try reproduce these observations in vivo. This relies on the assumption that bronchial epithelial cells isolated from control or asthma patients respond in the same way, which at this time we cannot answer. Similarly, the beneficial effects of AZM have been observed in respiratory disease patients again making the assumption that control/disease cells behave the same without foundation. Therefore, these data can be considered preliminary in nature and do not support or refute the effects of AZM observed in the in vitro experiments. Overall, AZM was well tolerated during this short study, as previously reported (Hahn et al. 2012), and while not a primary objective of the study, it is important to note that 4/10 asthma patients had an improvement in asthma symptoms (Asthma Control Questionnaire [ACQ] using previously reported thresholds [Juniper et al. 2005]). These data also illustrate the heterogeneity in the patient responses to AZM. However, overall, we did not observe similar effects on the bronchial epithelium as in vitro potentially due to the limitations of our study outlined.

This study is the first of its kind to directly relate laboratory findings with in vivo findings in relation to the epithelium in asthma. We have demonstrated for the first time that AZM significantly improves the development of a normal bronchial epithelial barrier in vitro, mimicking reepithelization postinjury. Similarly, we demonstrated the effects on maintenance of a barrier and the ability of the epithelial barrier to repair postchallenge. AZM suppressed MMP‐9 release which correlated with barrier integrity, suggesting a putative mechanism. In the clinical arm of the study, we did not observe significant effects of AZM on the bronchial epithelium in vivo. Our current findings support the development of AZM derivatives that maintain the potentially useful “extended” properties of the drug, but lack antibiotic properties and also other strategies targeting airway epithelial integrity. This study also emphasizes the challenge of directly translating in vitro finding to patients and the need for larger in vivo mechanistic studies.

## Conflict of Interest

None declared.
